# tRNA methyltransferase homologue gene *TRMT10A* mutation in young adult‐onset diabetes with intellectual disability, microcephaly and epilepsy

**DOI:** 10.1111/dme.13024

**Published:** 2016-08-17

**Authors:** T. W. Yew, L. McCreight, K. Colclough, S. Ellard, E. R. Pearson

**Affiliations:** ^1^ Division of Cardiovascular and Diabetes MedicineMedical Research InstituteNinewells Hospital and Medical SchoolDundeeUK; ^2^ Department of MedicineNational University Health SystemSingapore; ^3^ Department of Molecular GeneticsRoyal Devon & Exeter NHS Foundation TrustExeterUK; ^4^ Institute of Biomedical and Clinical ScienceUniversity of Exeter Medical SchoolExeterUK

## Abstract

**Background:**

A syndrome of young‐onset diabetes mellitus associated with microcephaly, epilepsy and intellectual disability caused by mutations in the tRNA methyltransferase 10 homologue A (*TRMT10A*) gene has recently been described.

**Case report:**

We report two siblings from the fourth family reported to have diabetes mellitus as a result of a *TRMT10A* mutation. A homozygous nonsense mutation p.Glu27Ter in *TRMT10A* was identified using targeted next‐generation sequencing and confirmed by PCR/Sanger sequencing. Diabetes was diagnosed while the subjects were in their 20s and was characterized by insulin resistance. Epilepsy and intellectual disability were features in common. Mild microcephaly was present at birth but their final head circumferences were normal.

**Conclusion:**

Our report provides independent confirmation of the role of *TRMT10A* mutations in this syndrome and expands its phenotypic description. *TRMT10A* sequencing should be considered in children or adults with young‐onset diabetes who have a history of intellectual disability, microcephaly and epilepsy. This report also shows the advantages of using a targeted panel to identify previously unsuspected monogenic diabetes among young‐onset non‐insulin‐dependent diabetes in the absence of obesity and autoimmunity.

## What's new?

- This report describes the fourth family reported to have diabetes mellitus caused by a mutation in the *TRMT10A* gene.
- In contrast to the previous families, the diabetes mellitus in the patients we describe was diagnosed after 20 years of age, and at diagnosis they had only mild learning disability and epilepsy with minimal other clinical features.
- *TRMT10A* sequencing should be considered in children or adults with young‐onset diabetes who have a history of intellectual disability, microcephaly and epilepsy.
- A targeted panel may be useful to identify previously unsuspected monogenic diabetes among individuals with young‐onset diabetes.

**What's new?**

- This report describes the fourth family reported to have diabetes mellitus caused by a mutation in the *TRMT10A* gene.
- In contrast to the previous families, the diabetes mellitus in the patients we describe was diagnosed after 20 years of age, and at diagnosis they had only mild learning disability and epilepsy with minimal other clinical features.
- *TRMT10A* sequencing should be considered in children or adults with young‐onset diabetes who have a history of intellectual disability, microcephaly and epilepsy.
- A targeted panel may be useful to identify previously unsuspected monogenic diabetes among individuals with young‐onset diabetes.

## Introduction

Recently, a novel syndrome of young‐onset diabetes mellitus or abnormal glucose homeostasis associated with microcephaly, epilepsy and intellectual disability attributable to homozygous mutations in the tRNA methyltransferase 10 homologue A (*TRMT10A*) gene was reported in two families 1, 2. In another report, an individual with *TRMT10A* deletion with failure to thrive, delayed puberty, intellectual disability and diabetes was described 3.

In the present paper, we report two siblings with young adult‐onset diabetes, associated with intellectual disability, microcephaly in childhood and epilepsy, as a result of a third homozygous mutation in the *TRMT10A* gene.

## Case report

**Figure 1:**
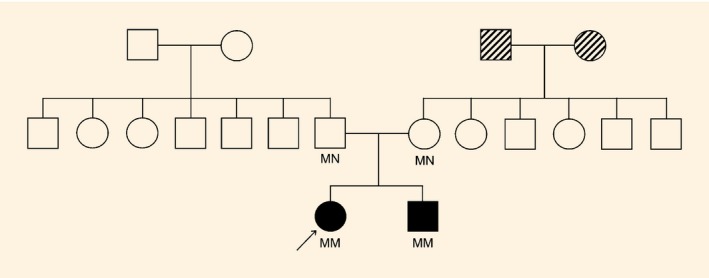
Family pedigree. Filled symbols denote individuals with young‐onset diabetes, childhood microcephaly, epilepsy and intellectual disability; hatched symbols denote relatives with adult‐onset diabetes; arrow denotes the proband. The mutation status (MN, heterozygous *TRMT10A* p.Glu27Ter mutation; MM, homozygous *TRMT10A* p.Glu27Ter mutation) is shown under each symbol.

The proband was the first child born to non‐consanguineous white parents (Fig. 1). The pregnancy was uneventful and the child was born at term without postnatal problems. The child's birth weight was 2700 g (‐1.6 sd), length was 47.2 cm (‐1.7 sd) and she had mild microcephaly [head circumference 32.5cm (‐2 sd)]. She had mild intellectual disability, first noticed when she attended primary school, but other developmental milestones were normal. She was diagnosed with grand‐mal epilepsy at 5 years old. MRI of the brain was normal. Her head circumference was 49 cm (‐2 sd) at 6 years 9 months old but improved to 50.3 cm (‐1.3 sd) at 9 years 5 months old. Her final head circumference was normal at 53.1 cm (‐1.3 sd). Growth in terms of height and weight was normal. Diabetes was diagnosed at 24 years old [plasma glucose 25.1 mmol/l, HbA_1c_ 142 mmol/mol (15.1%), BMI 24.2 kg/m^2^ (weight 62.8 kg, height 1.61 m)]. She had a buffalo hump but features of familial partial lipodystrophy and Cushing's syndrome were absent. She did not have ketoacidosis and was negative for antiglutamic acid decarboxylase and anti‐islet antigen‐2 antibodies. She was treated with insulin (1.0–1.2 units/kg/day) and metformin. Her HbA_1c_ ranged from 50 to 85 mmol/mol (6.7 to 9.9%). Fasting C‐peptide, measured 8 years after diagnosis, was detectable at 540 pmol/l. She had severe preproliferative retinopathy at 4 months after diagnosis, suggestive of long‐standing hyperglycaemia, but has no nephropathy or neuropathy to date.

The proband's brother was born at 43 weeks gestation. Microcephaly was described at birth but the head circumference was not recorded. His birth weight was 3274 g (‐0.7 sd). He started walking at 3 years old and could first speak in phrases at 6 years old. He attended special school because of intellectual disability. Epilepsy was diagnosed at 4 years old and he had delayed puberty. His final head circumference was normal at 54.5 cm (‐0.4 sd). Diabetes was diagnosed when screened at 28 years using a 75‐g oral glucose tolerance test (OGTT; plasma glucose 8.8 mmol/l at 0 min, 19.8 mmol/l at 120 min). His HbA_1c_ level was 60 mmol/mol (7.6%) and his BMI was 28.4 kg/m^2^ (weight 89.0 kg, height 1.77 m). Physical examination was normal. Fasting C‐peptide level was 1000 pmol/l. The HbA_1c_ level improved to 43 mmol/mol (6.1%) after 3 months of metformin therapy.

Neither the proband nor her brother had spontaneous hypoglycaemia. Both of them had mildly elevated LDL cholesterol and normal liver function tests. Skeletal surveys showed attenuated frontal skull vaults in both of them, in keeping with the history of microcephaly, but epiphyseal dysplasia was absent. Both their parents had normal heights, head circumferences and BMI, no epilepsy, intellectual disability, diabetes or prediabetes (normal OGTT results). Their maternal grandparents, now deceased, had Type 2 diabetes diagnosed after their 60s; their genotypes are not known.

All subjects provided written informed consent for blood sample collection and studies, as well as for the writing and publication of this report. The proband had participated in the UNITED (Using Pharmacogenetics to Improve Treatment in Early‐Onset Diabetes) study when analysis of her *HNF1A* and *HNF4A* genes did not identify any mutation. Testing for mutations in all of the known or putative monogenic diabetes genes as part of the study was undertaken using targeted next‐generation sequencing as previously described 4. Sequencing was performed with a HiSeq2000 system (Illumina, San Diego, CA, USA; 48 samples per lane) and 100 bp paired end reads. Mutation confirmation was performed by PCR/Sanger sequencing. Subsequently, samples of the proband's brother and parents were tested for *TRMT10A* mutation using PCR/Sanger sequencing. Plasma glucose and insulin was measured in the proband's brother and parents at 0, 30, 60, 90 and 120 min in the 75‐g OGTT. As a comparator, surrogate indices for insulin resistance and β‐cell function of the brother were compared with the means of four age‐ and BMI‐matched healthy control subjects.

## Results

### Metabolic studies

**Figure 2:**
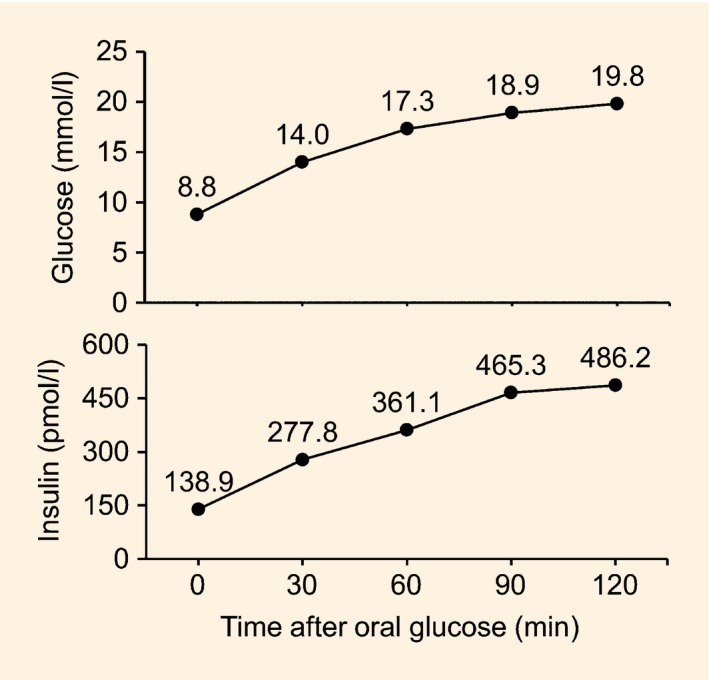
Results of 75‐g oral glucose tolerance test with plasma glucose and insulin measurements in the proband's brother, who was previously not known to have diabetes.

The OGTT results of the proband's brother are shown in Fig. 2. He was newly diagnosed with diabetes. The findings were suggestive of insulin resistance [Matsuda index 1.46; homeostatic model assessment of insulin sensitivity (HOMA%S) 34.7]^5^, ^6^, ^7^ and impaired β‐cell function (insulinogenic index 0.21; disposition index 0.32; homeostatic model assessment of β‐cell function (HOMA%β) 63.6]^6^, ^7^, ^8^. For comparison, the mean (sd) values for the four age‐ and BMI‐matched controls were: Matsuda index 14.2 (9.5); HOMA%S 165 (114); insulinogenic index 0.99 (0.52); disposition index 12.70 (11.85); HOMA%β 106 (51). The Matsuda indices of the father and mother were 8.86 and 10.49, respectively.

### Genetic studies

A homozygous G to T transition in exon 2 of gene *TRMT10A* at nucleotide position 79 of the coding sequence was identified in the proband. This variant replaces a glutamic acid residue with a premature termination codon at position 27 of the polypeptide (NM_001134665.1: c.79G>T; p.Glu27Ter). Mutations of other monogenic diabetes genes were not detected. This mutation was confirmed on Sanger sequencing to be homozygous in the proband and her brother, and heterozygous in both parents.

## Discussion

Igoillo‐Esteve *et al*. 1 first described a new syndrome of young‐onset diabetes, microcephaly, intellectual disability and epilepsy attributable to a homozygous nonsense mutation p.Arg127Ter in *TRMT10A* in a consanguineous family of Moroccan origin, with three out of nine children affected. Gillis *et al*. 2 identified a missense mutation p.Gly206Arg in *TRMT10A* in three out of 12 siblings born to non‐consanguineous parents from a small, inbred Jewish community in Uzbekistan. More recently, Zung *et al*. 3 described an individual with *TRMT10A* deletion with failure to thrive, delayed puberty, intellectual disability and diabetes. In the present paper, we report the eighth and ninth individual from the fourth family known to have this syndrome as a result of a *TRMT10A* mutation.

The clinical characteristics of our patients are compared with previous reports in Table 1. Diabetes caused by *TRMT10A* mutations was diagnosed between the ages of 9 and 28 years; two siblings did not have diabetes at the age of 13 and 14 years old. Interestingly, all three siblings in the family described by Gillis *et al*. 2 had hypoglycaemia, as did the individual reported by Zung *et al*. 3; however, no hypoglycaemia was reported in our patients or in those reported by Igoillo‐Esteve *et al*. 1. The individuals in the report by Gillis *et al*. and in our family did not have high birth weight, suggesting intrauterine hyperinsulinaemia was not present. Insulin resistance appeared to be the dominant pathophysiological mechanism in our patients. This was shown by the OGTT results in our proband's brother which were similar to those of the first individual described by Gillis *et al*. Similarly to the individuals reported by Igoillo‐Esteve *et al*., the insulin requirement of our proband was quite high. Based on the OGTT results, the parents who had heterozygous mutation did not appear to be at increased risk of diabetes.

**Table 1:** Clinical characteristics of patients with reported *TRMT10A* mutations

Report [reference]	Family 1			Family 2			Family 3	Family 4	
	Igoillo‐Esteve *et al*. 1			Gillis *et al*. 2			Zung *et al*. 3	Present report	
Individual designation	Inidividual 1	Individual 2	Individual 3	Individual 4	Individual 5	Individual 6	Individual 7	Individual 8	Individual 9
Parental origin	Moroccan	Moroccan	Moroccan	Jewish	Jewish	Jewish	Israeli Muslim	Caucasian	Caucasian
Consanguinity between parents	Yes	Yes	Yes	No	No	No	Yes	No	No
*TRMT10A* mutation	c.379G>A p.Arg127Ter	c.379G>A p.Arg127Ter	c.379G>A p.Arg127Ter	c.616G>A p.Gly206Arg	c.616G>A p.Gly206Arg	c.616G>A p.Gly206Arg	4q23 deletion	c.79G>T p.Glu27Ter	c.79G>T p.Glu27Ter
Diabetes mellitus	Yes	Yes	Yes	Yes	No	No	Yes	Yes	Yes
Age when diabetes diagnosed (years)	22	19	14	9	–	–	15	24	28
Diabetes treatment	Insulin	Insulin	Insulin	Diet	–	–	Insulin	Insulin, metformin	Metformin
Endogenous insulin secretion	Detectable C‐peptide	Detectable C‐peptide	Detectable C‐peptide	Present but insufficient relative to insulin sensitivity	Inappropriately high during hypoglycaemia	Inappropriately high during hypoglycaemia	Detectable C‐peptide	Detectable C‐peptide	Present but insufficient relative to insulin sensitivity
Microcephaly at birth	Unknown	NR	No	Yes, mild	Yes, mild	Yes, mild	Yes, severe	Yes, mild	Yes
Microcephaly persistent	Yes, severe	Yes, severe	Yes, severe	NR	NR	NR	Yes, severe	No	No
Low birth weight	Unknown	NR	NR	Yes	Yes	Yes	Yes	No	No
Short stature	Yes	Yes	Yes	Yes	Yes	Yes	Yes	No	No
Epilepsy	Yes	NR	NR	Yes	Yes	Yes	No	Yes	Yes
Intellectual disability	Yes	Yes	Yes	Yes	Yes	Yes	Yes	Yes	Yes
Spontaneous hypoglycemia	NR	NR	NR	Yes	Yes	Yes	Yes	No	No
Brain imaging	Normal	NR	NR	Normal	Normal	NR	Normal	Normal	Unknown
BMI (kg/m^2^)	26.9	21.7	20.6	NR	NR	NR	18.2	24.2	28.4
Delayed puberty	NR	NR	NR	Yes	NR	NR	Yes	No	Yes
Other clinical features	Short neck, wide nose, low hairline, buffalo hump, retraction of right 5^th^ toe, scoliosis, joint laxity	NR	NR	NR	NR	NR	Small face, clinodactyly, sensorineural hearing impairment	Buffalo hump	No

NR, not reported. Mutations described in accordance with Human Genome Variation Society (HGVS) guidelines with the A of the ATG initiation codon numbered nucleotide c.1, using reference sequence NM_001134665.1 for *TRMT10A*.

Microcephaly was a feature in common to all the reported cases, but the degree of severity appeared to vary. Microcephaly is defined as an occipito‐frontal head circumference of >2 sd or >3 sd below the mean for age and sex 9, 10. Similar to those reported by Gillis *et al*. 2, our patients just met the criteria for mild microcephaly at birth; however, their head circumferences normalized as they grew and achieved normal final head circumferences. This was in contrast to the more marked microcephaly, which persisted to adulthood in the individuals reported by Igoillo‐Esteve *et al*. 1 and Zung *et al*. 3. All the individuals had intellectual disability, and epilepsy was common except in the individual reported by Zung et al. Contrary to the previous reports, our patients did not have short stature, although the proband did have a buffalo hump, as described in one patient previously 1.

A homozygous nonsense mutation, p.Glu27Ter, in the *TRMT10A* gene was identified in our patients. Data from the Exome Aggregation Consortium (ExAC) browser showed that the frequency of heterozygous *TRMT10A* nonsense mutations is ~1 in 4000, and the p.Glu27Ter mutation found in our family is the most common 11. The family is unaware of any close common ancestor. In humans, TRMT10A is the orthologue most closely related to yeast TRM10, a protein that has tRNA m^1^G_9_ methyltransferase activity 12. This nonsense mutation in *TRMT10A* at this location is likely to result in nonsense‐mediated decay and reduced protein expression 13. Reduced TRMT10A mRNA expression and TRMT10A protein deficiency was previously shown to be the result of a p.Arg127Ter nonsense mutation 1. TRMT10A protein is ubiquitously present but more abundant in human brain and pancreatic islets 1. This was consistent with the selected involvement of brain (microcephaly, intellectual disability and epilepsy) and pancreatic islets (diabetes) in our patients. The robust insulin secretion in our case supported the *in vitro* findings that TRMT10A silencing did not appear to affect β‐cell function but may induce β‐cell apoptosis 1. The mechanism by which the mutation is associated with insulin resistance remains to be investigated.

In summary, the present case report provides independent confirmation of the role of *TRMT10A* mutations in this newly described syndromic form of monogenic diabetes and expands its phenotypic description. It also highlights the advantages of using a targeted panel to identify previously unsuspected monogenic diabetes in young‐onset non insulin‐dependent diabetes in the absence of obesity and autoimmunity. Children or adults with young‐onset diabetes who have intellectual disability, microcephaly and epilepsy should undergo genetic testing for *TRMT10A* mutations. Further studies are needed to evaluate the prevalence of *TRMT10A* mutations in this phenotypic group.

## Funding sources

This work was undertaken as part of the UNITED study, funded by the Health Innovation Challenge Fund (HICF), a parallel funding partnership between the Wellcome Trust and the Department of Health (grant no. 091985/HICF 1009‐041). T.W.Y. is supported by an Academic Medicine Development Award from National University Health System, Singapore. S.E. is a Wellcome Trust Senior Investigator. E.R.P. holds a Wellcome Trust New Investigator award.

## Competing interests

None declared.
